# Bone turnover markers can predict healing time in medication-related osteonecrosis of the jaw

**DOI:** 10.1007/s00520-021-06361-z

**Published:** 2021-06-30

**Authors:** Lorenz Schubert, Guenter Russmueller, Heimo Lagler, Selma Tobudic, Elisabeth Heindel, Michael Kundi, Christoph Steininger

**Affiliations:** 1 Department of Medicine I, Division of Infectious Diseases and Tropical Medicine, Medical University Vienna, Waehringer Guertel 18-20, 1090 Vienna, Austria; 2 Department of Oral and Maxillofacial Surgery, Medical University Vienna, Vienna, Austria; 3 Center for Public Health, Medical University Vienna, Vienna, Austria

**Keywords:** Medication-related osteonecrosis of the jaw, Actinomycosis, Prediction of healing duration, Personalized medicine

## Abstract

**Objectives:**

Medication-related osteonecrosis of the jaw (MRONJ) is a severe and difficult-to-treat adverse event of bone-modifying agents. Therefore predictive strategies determining patients at risk for a prolonged healing duration are needed to optimize treatment. Thus, the present study evaluates whether or not bone turnover markers can be used to predict the healing duration in MRONJ patients.

**Materials and methods:**

The present study is a retrospective data analysis of patients suffering from MRONJ and positive histology for *Actinomyces* spp., who were identified at the General Hospital Vienna from 2014 to 2018. During the first visit, the patients’ demographics and levels of bone formation parameters were compiled. Healing times were analysed by Cox regression in dependence on these factors.

**Results:**

A total of 52 patients were identified who fulfilled the inclusion criteria. The indication for bone-modifying agents was breast cancer (n = 21), prostate cancer (n = 14), multiple myeloma (n = 6) and other malignant diseases (n = 11). In 43 (82.7%) of our patients, we were able to document complete mucosal healing. Furthermore, patients who responded faster to therapy showed higher levels of C-telopeptide (P < 0.05), osteocalcin (P < 0.05) and bone-specific alkaline phosphatase (P < 0.05), but lower levels of 1.25-dihydroxyvitamin D (P < 0.05) than slower responding patients. No correlation was found regarding parathyroid hormone or calcitonin levels. Interestingly, patients who had a slower response were less likely to report dental procedures, but more likely to report a history of chemotherapy.

**Conclusion:**

CTX and osteocalcin levels may be used for predicting healing duration for MRONJ.

## Introduction

Malignant bone disease is common in many malignancies, such as breast cancer, prostate cancer and multiple myeloma [1]. Bone-modifying agents (BMA), which suppress bone resorption due to metastasis, became an important option in oncological treatment. However, it is associated with the risk for severe complications, such as the medication-related osteonecrosis of the jaw (MRONJ). Incidence rates range between 0.1 and 10%, depending on the treatment dose as well as underlying disease, and its occurrence is often triggered by dental procedures [2].

MRONJ manifests as an osteonecrosis of mandibula or maxilla, recognizable by exposed bone, which persists for more than 8 weeks and is associated with a recent history of BMA in the absence of radiation therapy. As MRONJ is associated with an increased morbidity and reduced quality of life, an effective treatment protocol is crucial. Current guidelines recommend the discontinuation of BMA and a combination of antibiotic and surgical treatment [2]. Nevertheless, high rates of recurrences and failures have been reported [3].

Multiple factors play a role in MRONJ development. BMA are widely used in preventing skeletal-related events and to prolong survival in patients with breast, lung, and prostate cancer, multiple myeloma, Paget’s disease and osteoporosis. BMA generally comprise bisphosphonates and denosumab. Bisphosphonates suppress bone remodelling by inhibition of osteoclast differentiation, osteoclast function and increased apoptosis. Furthermore, a direct cytotoxic and antiangiogenic effect has been proposed [2, 4]. Denosumab is an inhibitor of receptor-activated nuclear factor kappa-B ligand (RANKL), a mediator of osteoclast differentiation. It is known to reduce the incidence of skeletal-related events (SRE) and delays SRE more effectively then bisphosphonates. However, the meta-analysis of Qi et al. indicated a potential higher risk for MRONJ development compared to bisphosphonates [5]. Interestingly, Russmueller et al. found that a large majority (89%) of MRONJ lesions were infected by *Actinomyces* spp*.* [6]. Actinomycetes are gram-positive, non-spore-forming, anaerobic and microaerophilic bacterial microorganisms, which are part of the physiological oral flora. Nevertheless, in case of micro trauma, they can invade deep tissue structures and cause difficult-to-treat bone-infiltrating infections [7]. Furthermore, many co-factors were found to be associated with MRONJ, such as diabetes, smoking, dental extraction and other concurrent medication [8]. These conflicting results emphasise the multifactorial genesis of MRONJ.

Personalized medicine is increasingly employed across many areas of clinical practice, aiming to tailor treatment approaches to individual patients [9]. Given the high disease burden, high rates of recurrences and treatment failures of MRONJ, optimized treatment strategies are urgently needed. Bone turnover markers (BTM), describing the metabolic profile of the bone, have been frequently used to predict the risk for bone diseases such as osteoporosis, bone metastasis and Paget’s disease [6, 7]. Previous attempts to determine the usefulness of said biomarkers showed controversial results [10–16]. However, studies mainly focused on predicting occurrence of MRONJ, but have not examined the potential use of BTM to predict the risk for prolonged healing durations. BTM could provide urgently needed information at an early stage of treatment to personalise disease management [17].

Thus, the current study aimed to evaluate the predicate value of C-terminal telopeptide of type I collagen (CTX), osteocalcin (OC), bone-specific alkaline phosphatase (BAP), calcitonin, 25-hydroxyvitamin D, 1.25-dihydroxyvitamin D or parathyroid hormone, for the healing time of MRONJ.

## Material and methods

### Subjects

This retrospective study exclusively comprises patients suffering from MRONJ, treated at the Division of Infectious Diseases and Tropical Medicine and at the Department of Oral and Maxillofacial Surgery at the Medical University of Vienna, Austria, between September 2014 and September 2018. The study protocol was approved by the Ethics Committee of the Medical University of Vienna, Austria (ECS 2055/2016), and all study-related procedures were conducted according to the declaration of Helsinki. Diagnosis of MRONJ was established in concordance with the current guidelines of the American Association of Oral and Maxillofacial Surgeons (AAOMS) [2]. Inclusion criteria for this study were a clinically diagnosed MRONJ, histological evidence of *Actinomyces*, the availability of laboratory parameters describing the patient’s bone metabolism and no contraindication for surgical treatment. Patients were excluded when having received radiotherapy, declined the suggested antibiotic or surgical treatment for personal reasons, or when laboratory parameters were not available.

### Data collection

Demographic data regarding age, sex, height, weight, type of BMA, stage of MRONJ, history of chemotherapy and history of dentoalveolar procedures were retrospectively extracted from the Vienna General Hospital Information System (AKIM). BMA were separated by type of BMA and dosing. BMA were categorized high-dose if they were used for malignant disease and low-dose if they were used for osteoporosis or other diseases [18]. The extraction date of laboratory parameters was defined as the starting point of the present study. The values of osteocalcin (electrochemical luminescence immunoassay, ECLIA), BAP (chemical luminescence immunoassay, CLIA), CTX (ECLIA), calcitonin (ECLIA), parathyroid hormone (ECLIA), 1.25-dihydroxyvitamin D (CLIA) and 25-hydroxyvitamin D (CLIA), which were measured at the patient’s first presentation at our department, were recorded. The healing process was extracted from the patient history of the Department of Oral and Maxillofacial Surgery. The date of complete mucosal healing of MRONJ, which persisted for more than 4 weeks, was set to be the endpoint of the study. The healing duration was then calculated between starting and endpoint in weeks. Further the healing duration was assessed depending on the risk factors.

### Treatment procedures

MRONJ was categorized according to the AAOMS guidelines from stage 0 to stage 3 [2]. The aim of treatment was to achieve complete mucosal healing. Depending on the stage, patients were treated according to the standardised protocol. All patients received systemic antibiotic treatment with penicillin V (1000 IE tid), amoxicillin/clavulanic acid (875/125 mg tid) or cephalexin (1 g tid) and were, if possible, assigned to surgical treatment. Patients who reported an allergic reaction to β-lactam antimicrobial agents received doxycycline (100 mg bid) [6, 19–22]. Antimicrobial treatment was revaluated after minimum treatment duration of 4 months, depending on mucosal healing status. Further indication for BMA was closely revaluated with the attending oncologist and discontinued if possible. Surgical interventions were performed according to AAOMS. Patients were treated with local debridement for control of infection for stage 2 MRONJ and with extensive debridement for stage 3 MRONJ [2]. However, we additionally performed minimal invasive surgical interventions such as re-movement of necrotic bone and local revitalisation of the necrotic areas in stage 1 MRONJ to further improve the situation.

### Statistical analysis

Healing times were analysed by Cox regression. Patients that had not healed until the last follow-up date were censored at this date. Due to their skewed distribution, lab data were log-transformed. For illustrative purposes, statistically significant predictors were split at the median, and Kaplan–Meier plots were computed. For all statistical tests p-values below 0.05 were considered significant. Analyses were done by Stata 13.1 (StataSoft, USA), and graphs were produced by Statistica 10 (StatSoft, USA).

## Results

During the study duration, a total of 52 patients were identified who fulfilled the inclusion criteria. The demographic characteristics of the study population are reported in Table 1. The median age of these patients was 74 (63.5–78) years, and female and male patients were nearly equally divided (female, n = 27 [51.9%]). In 43 (82.7%) of the patients, it was possible to document complete mucosal healing. Among the patients who did not show complete mucosal healing, three patients received a combination of surgical and antibiotic therapy, and six patients received antibiotic treatment only. Patients showed a median healing time of 20.7 (11.54–32.32) weeks. The most frequently used antibiotic was penicillin V (n = 45 [86.5%]), followed by amoxicillin/clavulanic acid (n = 3 [75.8%]) and cephalexin (n = 1 [1.9%] or doxycycline (n = 3 [5.8%]) for patients with reported allergic reactions. Antimicrobial agents were dosed as highlighted in the methods. In one patient doxycycline dose was increased to 300 mg q.d. due to obesity and aggravating infection. Surgical interventions comprised minimal invasive symptomatic therapy for all stages, incision or local debridement of soft tissue for infection control in stages 2 or 3, and bone resection for patients in stage 3.

**Table 1:** Patients**’** baseline characteristics

Patients characteristics		Study group (n** = **52)
Age (years)		74 (63.5–78)
Gender (n[%])	Male	25(48.1)
	Female	27 (51.9)
Risk factors (n[%])	Smoker	31 (59.6)
	Diabetes mellitus	7 (13.5)
Trigger factor (n[%])	Dental procedure	35 (67.3)
Underlying disease (n[%])	Breast cancer	21 (40.4)
	Prostate cancer	14 (26.9)
	Multiple myeloma	6 (11.5)
	Other malignant diseases	11 (21.2)
Bone-modifying agents (n[%])	Bisphosphonate	8 (15.4)
	Denosumab	31 (59.6)
	Combination	13 (25)
Chemotherapy (n[%])		35 (67.3)
AAOMS MRONJ stage ^a^ (n[%])	Stage 1	36 (69.2)
	Stage 2	8 (15.4)
	Stage 3	8 (15.4)
Treatment (n[%])	Surgical intervention and antibiotic treatment	42 (80.8)
	Only antibiotic treatment	10 (19.2)
Antimicrobial treatment (n[%])	Penicillin V	45 (86.5)
	Amoxicillin/ clavulanic acid	3 (5.8)
	Cefalexin	1 (1.9)
	Doxycycline	3 (5.8)

*MRONJ *medication-related osteonecrosis of the jaw, *AAOMS* American Association of Oral and Maxillofacial Surgeons

^a^MRONJ stage was categorized according to the American Association of Oral and Maxillofacial Surgeons[2]

In our study population, 31 (59.6%) of the patients were smokers, and 35 (67.3%) patients had undergone tooth extraction prior to first signs of MRONJ. Eight patients (15.4%) received bisphosphonates, 31 patients (59.6%) received denosumab, and 13 patients (2.5%) received both options in the past. Mean duration of BMA was 2343 (863–4021) days in the bisphosphonate group, 609 (476–1037) days in the denosumab group and 979 (702–1054) days in the group with both treatment options. At admission BMA was discontinued in most patients (n = 47 [90.4%]). Additional five of the patients received an mTOR inhibitor, five patients received lenalidomide, two patients received a tyrosine kinase inhibitor, and two patients received a monoclonal VEGF antibody. Breast cancer was the most frequent reason for BMA (n = 21 [40.4%]), followed by prostate cancer (n = 14 [26.9%]), multiple myeloma (n = 6 [11.5%]) and other malignant diseases (n = 11 [21.2%]]. MRONJ stage 1 (n = 36 [69.2%]) was observed most frequently.

**Fig. 1:**
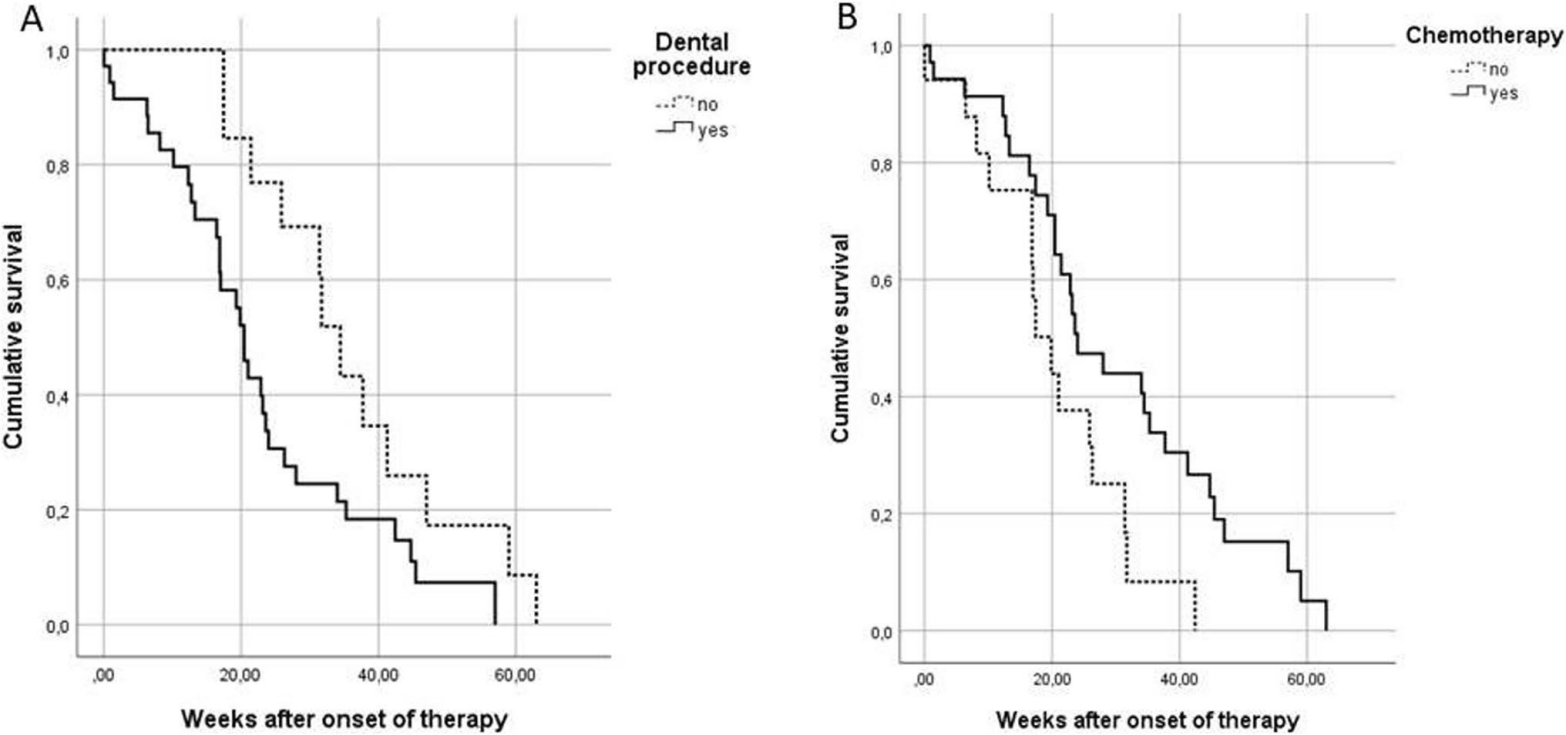
The Kaplan–Meier plots illustrate the healing duration of MRONJ depending on history of dental procedures (**A**) or chemotherapy (**B**)

The demographic and clinical parameters including smoking, history for diabetes mellitus, dental procedures, chemotherapy and BMA were further correlated to the required healing duration. The results are shown in Table 2. The analysis showed significantly faster healing durations for patients without chemotherapy (P < 0.05) and with a dental (P < 0.05) procedure in the past. Healing duration did not differ depending on the history of smoking or choice of BMA. The Kaplan–Meier blots are shown in Fig. 1. For the parameters smoking and BMA, no differences in healing duration were found.

**Table 2:** Duration of healing depending on the demographic parameters

Demographics	Status	Median (IQR) of weeks	*P* values
Smoking habit	Non-smokers	25.14 (12.79–36.07)	0.393
	Smokers	20.43 (16.86–34)	
Diabetes mellitus	no	22.14 (16.64–32.86)	0.393
	yes	20.43 (12.71–47)	
Dental procedures	No dental procedure	33.07 (23.64–44.14)	0.027
	Dental procedure	19.86 (12.29–26.29)	
Chemotherapy	No history of chemotherapy	17.43 (10.14–26.29)	0.033
	History of chemotherapy	23.36 (16.93–39.5)	
Bone-modifying agents	Bisphosphonate	17.14 (13.29–17.43)	0.84
	Denosumab	24.5 (16.93–36.07)	
	Both	23.57 (10.14–35.29)

**Fig. 2:**
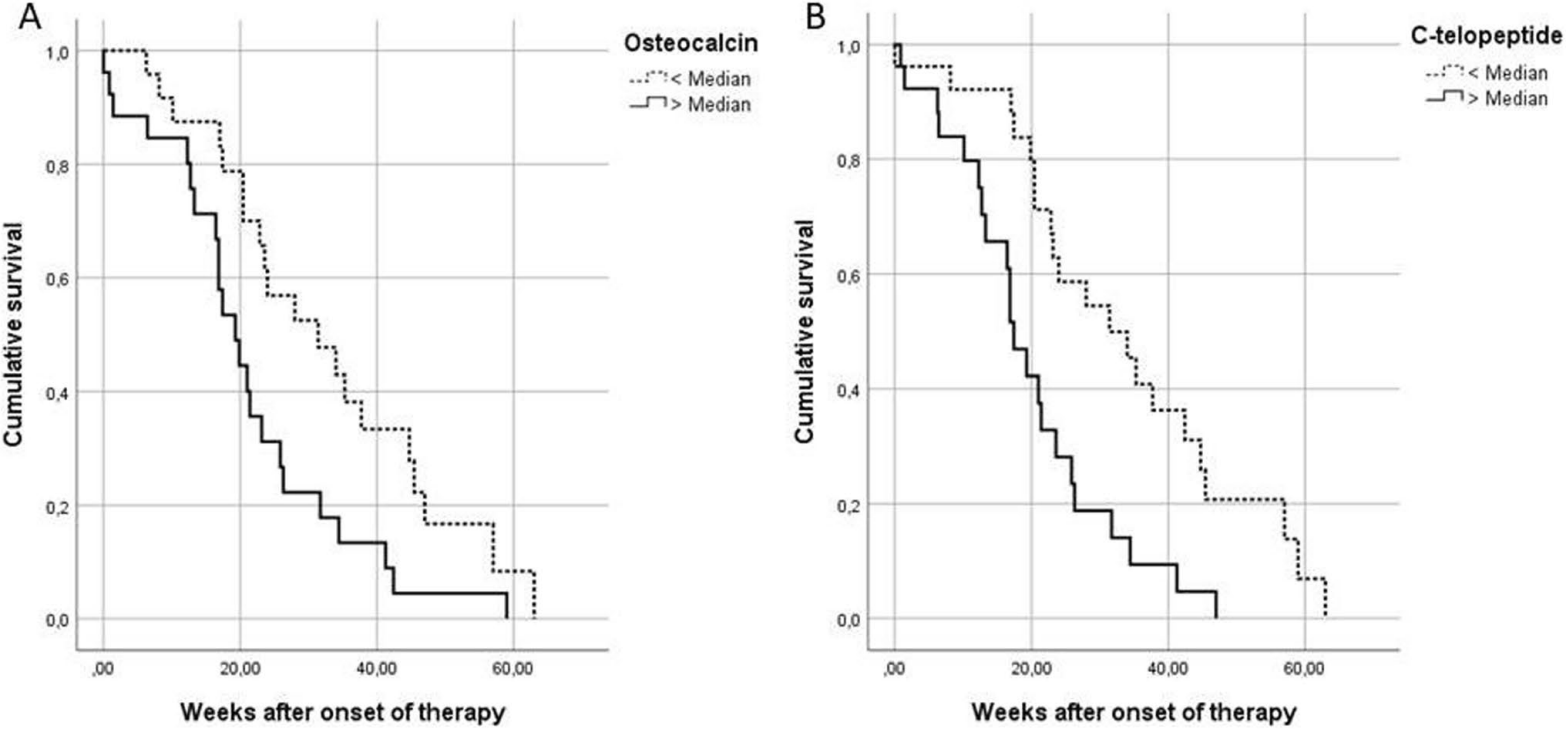
The Kaplan–Meier plots illustrate the healing duration of MRONJ depending on the median bone turnover marker levels osteocalcin (**A**) or C-telopeptide (**B**)

The bone turnover and hormone parameters, measured at the patients’ first visit, were then correlated to the healing duration in weeks needed. An overview of the healing duration depending on the laboratory parameters is illustrated in Table 3. The analysis showed significantly faster healing durations for patients with higher CTX levels (P < 0.05), higher osteocalcin levels (P < 0.05), higher BAP (P < 0.05) and lower 1.25-dihydroxyvitamin D levels (P < 0.05). For CTX and OC Kaplan–Meier blots were computed, as shown in Fig. 2. Concerning the hormone parameter levels of calcitonin and parathyroid hormone, no differences in healing duration were found.

**Table 3:** Comparison of healing durations in weeks for MRONJ below and above median for laboratory parameter levels

Laboratory parameter	Median	Median of weeks < median	Median of weeks > median	*P* values
C-telopeptide	110 pg/ml	28 (20.43–42.43)	17.14 (12.29–25.86)	0.001
Osteocalcin	9.95 ng/ml	26 (18.93–41.21)	19.29 (12.71–26.29)	0.021
BAP	8.2 ng/ml	21.64 (13.29–42.43)	21.43 (16.86–31.71)	0.016
Parathyroid hormone	48.5 pg/ml	20.43 (13.29–31.43)	24.93 (17.14–34.86)	0.707
Calcitonin	1.4 pg/ml	18.36 (12.5–24.71)	25.86 (17.43–41.29)	0.279
25-Hydroxyvitamin D	68.1 nmol/l	22.07 (10.14–35.29)	21.43 (17.43–34)	0.33
1.25-Dihydroxyvitamin D	49 pg/ml	17.43 (10.14–31.43)	23.79 (19.86–41.29)	0.008

*BAP* bone-specific alkaline phosphatase

## Discussion

This study was conducted to investigate the predictive potential of CTX, OC, BAP, parathyroid hormone, calcitonin, 1.25-dihydroxyvitamin D and 25-hydroxyvitamin on healing duration of MRONJ. Indeed we found significantly higher levels of CTX and OC, but lower levels of 1.25-dihydroxyvitamin D in patients who responded faster to standard treatment. The results suggest that longer healing periods of MRONJ occur in patients with extensive impairment of bone metabolism.

CTX, OC and BAP are considered conclusive markers for bone remodelling. Previously, they were mainly evaluated as predictive parameters for the development of MRONJ. Marx et al. described different risk groups in dependence on the CTX level after dental procedures. The study defined high risk for patients with CTX levels below 100 pg/ml, intermediate risk for patients with CTX levels between 100 and 150 pg/ml and low risk for patients with CTX levels above 150 pg/ml [16]. In contrast, a recent report from Hutechson et al. emphasised that CTX does not accurately predict risk for MRONJ on a case-by-case basis, as 177 patients in his cohort with CTX levels under 150 pg/ml did not develop MRONJ after tooth extraction [23]. Other biomarkers, such as OC or BAP, demonstrated little predictive correlation of MRONJ. The mean CTX level in our cohort was 170 pg/ml, which complies with the posed range for low risk of MRONJ [12, 16]. However, patients in our cohort suffered from oncological diseases which complicate the comparison, due to different BMA dosing schemata, co-administration of chemotherapy and impact of the disease itself. Further, CTX, OC and BAP were significantly lower in patients with longer healing duration in our cohort. Comparative data for BTM on disease severity is scarce. A study of 18 MRONJ patients described greater clinical symptoms in patients with lower CTX levels, measured by location and stage of MRONJ [11].

Inconsistencies of reported BTM risk ranges may reflect the multifactorial genesis of MRONJ. Therefore, we evaluated the influence of several factors, such as smoking habits, BMA, chemotherapy or dental procedures on the MRONJ outcome. One finding was that chemotherapy was associated with a longer healing duration. Chemotherapy is often long-lasting, consuming and accompanied by severe side effects. It is not surprising that an efficient MRONJ therapy, often consisting of antibiotics and surgery, is partly postponed if patients receive chemotherapy. Further antiangiogenic drugs, such as tyrosine kinase inhibitors and monoclonal antibodies targeting the vascular endothelial growth factor, were shown to worsen MRONJ by inhibiting bone remodelling, antagonising mucosal healing and exposing to infections during treatment [2]. The second finding was that treatment duration lasted longer in patients without past dental procedures. We believe that this reflects a more severe impairment of bone metabolism as MRONJ developed without direct dental trauma. The used BMA had no influence on the needed healing durations; however, mean healing duration was lower in the bisphosphonate group.

Previous studies emphasised an influence of 25-hydroxyvitamin D, parathyroid hormone and calcitonin on MRONJ development [10, 24, 25]. We found significantly lower levels of 1.25-dihydroxyvitamin D and a trend for lower 25-hydroxyvitamin levels in fast responders. In our view, low vitamin D levels are not a protective factor for slow healing of MRONJ, rather than an indicator for a higher requirement of vitamin D due to higher bone turnover in fast-healing patients. Although there is some data suggesting the involvement of hyperparathyroidism in MRONJ development, no significant correlation was found in the present study [10]. Calcitonin did not differ compared to the healing duration of MRONJ.

Treatment objectives of AAOMS are to eliminate pain, control infection and prevent disease progression [2]. In contrast aim of our treatment regimen was to achieve complete mucosal healing, in order to improve quality of life and to shorten intervals for re-administration of oncological treatments. Therefore, our treatment protocol was stricter than recommended by the AAOMS [2]. First, our surgical intervention protocol was stricter. As recommended by the AAOMS, patients received local debridement for control of infection in stage 2 and extensive debridement in stage 3 [2]. However, we performed minimal invasive surgical interventions, such as re-movement of necrotic bone and local revitalisation of the necrotic areas, to facilitate soft tissue healing in patients with MRONJ stage 1. Second, we opted for early antimicrobial treatment. The current guidelines consider antimicrobial treatment if clinical signs of infections are present [2]. In the present study all patients received antimicrobial therapy. This may seem to be overtreatment, but recently we demonstrated that a great number of histological bone samples (89%) were *Actinomyces* spp. positive [6]. Actinomycosis is a difficult to treat, chronic-progressive disease and requires antimicrobial treatment for several months in combination with surgery. In our experience, treatment response of ONJ was significantly better when actinomycosis was treated regardless of the stage of disease. Treatment of choice for actinomycosis is beta-lactam antibiotics or doxycycline for penicillin allergic patients. Antimicrobial treatment was continued for a minimum of four months, and then re-evaluated. If complete mucosal healing was established, the antimicrobial agent was discontinued. Finally, we closely evaluated indication for BMA and discontinued treatment if possible. The significance of BMA discontinuation is still debated, but there are some studies emphasizing a favourable surgical outcome [18, 26].

We did not use teriparatide, pentoxifylline, tocopherol or hyperbaric oxygen therapy in this cohort. Teriparatide, a recombinant human parathyroid hormone, was recently assessed to be beneficial in improving the rate of resolutions of MRONJ and in reducing the bony defect volume in comparison to placebo [27]. Concerns regarding the osteoanabolic mechanism of teriparatide leading to cellular proliferation and ultimately exacerbation of malignant bone disease were not supported by the study [27, 28]. However, information of long-term efficacy and safety are needed. Other investigational treatments such as pentoxifylline, tocopherol and hyperbaric oxygen were described to be beneficial, but their exact indication remains to be assessed [29, 30].

Some may criticise our strict selection of histological evidence of Actinomyces-positive MRONJ patients. However, the cohort of Actinomyces-negative samples was too small for separate analysis, and we wanted to minimize the risk of bias, regarding different treatment protocols depending on Actinomyces status.

We acknowledge several limitations. First, our investigation is limited by the variability of BTM, as it is also affected by multiple non-specific factors like age, sex, day-time, food intake and liver function. BTM represent bone resorption and bone formation in the body as a whole. Factors such as the activity and type of underlying disease, chemotherapy and especially glucocorticoids could have influenced the measured BTM levels, but could not be assessed in this retrospective study [1]. Second, this study is retrospective data analysis and dependent on precise documentation. Although patients at our centre were treated according to standardized treatment protocols, there were some factors, such as precise ending of antimicrobial treatment, which we could not assess retrospectively. Finally, our investigation is also limited by the small sample size due to low prevalence of MRONJ.

In conclusion, we found that level of bone metabolism, characterized by levels of CTX, OC, BAP and 1.25-dihydroxyvitamin D, is associated with time required for healing of MRONJ. This data could be used in future to tailor treatment approaches to individual patients. However, prospective studies with carefully matched groups and standardised measurement protocols are needed to validate their potential to increase treatment quality.

## Data Availability

Data is available on request from the corresponding author.
